# Supplementary material for: Counteracting structural errors in ensemble forecast of influenza outbreaks
Source: Nat Commun. 2017 Oct 13;8:925. doi: 10.1038/s41467-017-01033-1 (PMC5640637; doi:10.1038/s41467-017-01033-1)
Supplement: Supplementary file 1 — Supplementary Information [file 41467_2017_1033_MOESM1_ESM.pdf]

Supplementary Note 1. Error structure in SIRS model

An SIRS model with transmission potential modulated by observed humidity conditions has previously been used to explain the observed seasonal cycle of excess pneumonia and influenza mortality in the United States during 1972 to 2002 [1]. This humidity-driven SIRS model is a reliable, albeit simple, mathematical description of the transmission process for influenza. In the SIRS model, a population is sub-divided into three categories: those susceptible (S) to, infected (I) with, and recovered (R) from influenza. Suppose the total population is N, the contact rate at time t is $\beta(t)$, the average duration of immunity is L, the mean infectious period is D, and the rate of infection imported from external sources is α. The dynamical evolution of influenza within this population can then be described as:

$$\frac{dS}{dt} = \frac{N - S - I}{L} - \frac{\beta(t)IS}{N} - \alpha, \tag{1}$$

$$\frac{dI}{dt} = \frac{\beta(t)IS}{N} - \frac{I}{D} + \alpha. \tag{2}$$

Here, the contact rate $\beta(t)$ is related to absolute humidity (AH) through the basic reproductive number $R_0(t) = \beta(t)D$. Laboratory experiments indicate that $R_0(t)$ has an exponential relationship with humidity [2], which affects the survival and transmission of the influenza virus:

$$R_0(t) = \exp(a \times q(t) + b) + R_{0min}, \tag{3}$$

where $q(t)$ is daily observed specific humidity (a measure of AH), $a = -180$, $b = \log(R_{0max} - R_{0min})$, and R_{0max} and R_{0min} are the maximal and minimal daily basic reproductive number, respectively. In the following analysis, we fix the total population at $N = 500,000$ and the external import of infection at $\alpha = 0.1$ (one infection every 10 days), thus the state vector is $\mathbf{x}^t = (S(t), I(t), L, D, R_{0max}, R_{0min})$. In a realistic setting, the ranges of the model parameters are $2y \leq L \leq 10y$, $2d \leq D \leq 7d$, $1.3 \leq R_{0max} \leq 4$ and $0.8 \leq R_{0min} \leq 1.3$ [1].

For this non-autonomous dynamical system in which AH varies with time, it is difficult to analyze the exact error structure using linear techniques. As an alternative, we adopt the breeding method to inspect the relationship between errors in variables/parameters and observation I [3, 4]. Unlike linear approximation, the breeding method fully preserves the nonlinear dynamics of the system. In numerical weather prediction (NWP), it has been

used to estimate fast-growing directions of errors [3, 4]. In our study, thanks to the low dimensionality of the humidity-forced SIRS model, we are able to explore the detailed error structure by breeding a small perturbation on variables and parameters. Specifically, for a variable or parameter $x \in \mathbf{x}^t$ at time t, we impose small random perturbations on x, obtaining a perturbed state $\mathbf{x}_p^t$. Then both the unperturbed trajectory $\mathbf{x}^t$ and the perturbed $\mathbf{x}_p^t$ are integrated forward following the nonlinear dynamics (Equations 1-S2) for a period of time Δt. The bred error at time $t+\Delta t$ is calculated as the difference between the perturbed and unperturbed trajectories: $\delta \mathbf{x}^{t+\Delta t} = \mathbf{x}_p^{t+\Delta t} - \mathbf{x}^{t+\Delta t}$.

In order to produce a typical influenza outbreak, we use a combination of model parameters, $L = 3.86y$, $D = 2.27d$, $R_{0max} = 3.79$ and $R_{0min} = 0.97$, which generates representative seasonal cycles of 1972-2002 influenza outbreaks in New York State [1]. The SIRS model is initiated on October 1, 1972 with initial conditions $S(0) = 250,000$ and $I(0) = 1$, and is forced by observed daily AH for New York State. To inspect the error structure between the state variables/parameters S, R_{0max}, R_{0min}, D, L and I, we impose random perturbations on a specific state variable/parameter and the observed variable I at different phases of the outbreak. For example, for the combination (S, I) at 6 weeks, 3 weeks, 1 week prior to peak and 1 week after peak, we repeatedly add 1,000 random errors uniformly distributed in the region $[-20\%, +20\%] \times [-20\%, +20\%]$ to (S, I). The perturbed trajectories are then evolved according to the SIRS dynamics for one week, and the bred errors $(\delta S, \delta I)$ are calculated as the difference between the perturbed trajectories and the unperturbed.

In Fig. 1, we present the evolution of these random errors at different stages of SIRS spreading. The initial errors are displayed by red dots, while the bred errors after one week are indicated by blue dots. The solid red and blue lines highlight the cases for which initial perturbations were only imposed on the unobserved state variable/parameter. It is seen that, for perturbations imposed before the peak (Fig. 1A-C), errors in the observed state variable I for sensitive unobserved state variables/parameters such as S and R_{0max} expand quickly, and a clear nonlinear relationship for $(\delta S, \delta I)$ and $(\delta R_{0max}, \delta I)$ emerges. For R_{0min} and D, there also exists a nonlinear trend; however, the relationship is not clear enough to determine errors in R_{0min} and D as a function δI, as the observational error is large compared to the true value of I. For the least sensitive parameter L, the error has almost no relationship with errors in the observed variable. After the peak (Fig. 1D), while most errors decay due to the contracting dynamics of the SIRS model, the clear error structure

of $(\delta S, \delta I)$ and $(\delta R_{0max}, \delta I)$ remains.

We note that the smooth and continuous error manifold in Fig. 1 is constructed under the assumption that the system state is far from criticality in parameter space. However, it is well established that epidemic dynamics may evolve in the neighborhood of critical points that mark the onset of phase transitions. Around these points, even small perturbations in the state variables can produce significant qualitative changes in system dynamics. These changes may include abrupt transitions from low to high endemicity, oscillations and chaotic behaviors [5, 6]. Indeed, previous theoretical works have confirmed that oscillations and even chaos can be present in simple nonlinear epidemic models with periodic forcing [7–11]. Such complexity, confirmed by real-world observations, makes mathematical prediction of future infectious disease incidence challenging.

In this work, we focus on short-range forecasts in real-time during one influenza season. Therefore, extremely complex dynamical behaviors, such as oscillations and chaos, that appear over multiple outbreak cycles are not present. In other work, multiannual forecasts of qualitative features of seasonal influenza have been made using a metapopulation model that incorporates information on temperature, humidity, antigenic drift and immunity loss [12]. Over these time scales, error growth will saturate, due to system nonlinear dynamics, which prevents the one-to-one mapping between errors in the observations and unobserved state variables. As a consequence, the error breeding approach introduced here should only be applied to short-term predictions.

In a single influenza season, however, a second-order (or continuous) transition from an outbreak-free phase to an outbreak phase may occur. To examine the error structure around such transition points, we performed the same error breeding procedure for the maximum basic reproductive number, R_{0max}. Starting with the same initial conditions as in Fig. 1a in the main text, we varied the value of R_{0max} and found the critical point $R_{0max} \approx 3.0$ through simulations. We then imposed perturbations on R_{0max} to construct the error structure between R_{0max} and weekly incidence observations. As shown in Fig. 2, the error structure appears to be a step-function. This is because, below the critical value, the error in the observations is constant. In this case, the error structure can still be fitted using a 3rd-order polynomial after discarding the constant part, as shown in Fig. 2. This special circumstance, however, rarely exists in realistic influenza outbreaks.

Supplementary Note 2. Robustness of error structure

The above analysis is performed assuming that all variables/parameters but the perturbed are perfectly known. Such an assumption does not apply to real-world applications. In a more realistic setting, a natural question arises as to whether the error structure is stable against perturbations on other state variables. To answer this question, a robustness analysis of the error structure $(\delta S, \delta I)$ is performed as follows. At 3 weeks prior to peak, we impose uniformly distributed errors in the range $[-20\%, +20\%]$ to variable S, and then display the structural errors $(\delta S, \delta I)$ after one week of breeding. In order to explore the effect of perturbations on other state variables, additional shocks are applied to I, R_{0max}, R_{0min} and D separately 3 weeks before the peak. The magnitude of these shocks ranges from -15% to $+15\%$ with a 5% step. Finally we compare the original error structure and the ones with perturbed state variables in Fig. 3A. The same analysis can be applied to $(\delta R_{0max}, \delta I)$, $(\delta R_{0min}, \delta I)$ and $(\delta D, \delta I)$ (see Fig. 3B-D). Here we exclude the parameter L as we have already shown that system dynamics are insensitive to a change in L at these time scales.

In Fig. 3, the title of each panel indicates the state variable/parameter with the additional shock, and the structural errors of the y-axis variable/parameter as a function of these various additional shocks are displayed by different colored lines. We mark the $\pm 20\%$ error boundary for the observed state variable, I, with vertical dash lines for better reference. The error structure of the sensitive state variable S and parameter R_{0max} is quite robust against error applied to the other variables/parameters (top 2 rows of Fig. 3), especially for perturbations on I, R_{0min} and D. In contrast, for the relatively less sensitive state variables R_{0min} and D, the error structure is shifted dramatically by perturbations to the other variables/parameters (bottom 2 rows of Fig. 3). Moreover, the curves for R_{0min} and D incline to align with the y-axis when S bears errors (left column bottom 2 rows of Fig. 3), which makes for unreliable inference of the errors in R_{0min} and D from the errors in the observation. We repeated this analysis for other typical combinations at different phases of the outbreak and found that the observed robustness of error structure for S and R_{0max} remains. This finding implies that the error correction should be effective for S and R_{0max} even in the presence of error in other state variables and parameters.

Supplementary Note 3. Error correction in perturbed SIRS simulations

Given the clear nonlinear relationship between the errors in state variable S, parameter R_{0max} and the observed variable I, it is natural to use this error structure to diagnose existing errors in S and R_{0max}. In the following analysis, we will focus on variable S, though the same analysis on R_{0max} can be applied. For the nonlinear error structure $(\delta S, \delta I)$, observed in Fig. 1, we can fit the data points with a 3rd-order polynomial, as shown in Fig. 1a in the main text. The obtained curve can be used to infer the error δS in variable S from the error δI of the observed state variable relative to the observation. In applications, since δS and δI have different scales, it is convenient to normalize the bred errors by corresponding largest absolute values before conducting the curve fitting. This procedure helps to avoid a badly conditioned polynomial fit, in which the δI data points are clustered within a narrow region.

Algorithm 1 Error correction of perturbed SIRS trajectory

1: Input: State vector estimation at week $t-1$, $\boldsymbol{x}^{t-1} = (S^{t-1}, I^{t-1}, R_{0max}^{t-1}, R_{0min}^{t-1}, D^{t-1}, L^{t-1})^T$, observation I_{obs}^t at t week.

2: Breeding method: impose Gaussian-distributed random errors on S^{t-1} to form multiple perturbed trajectories $\boldsymbol{x}_p^{t-1}$, integrate both $\boldsymbol{x}^{t-1}$ and $\boldsymbol{x}_{\boldsymbol{p}}^{t-1}$ for one week.

3: The bred errors at t week are $\delta S = S_p^t - S^t$ and $\delta I = I_p^t - I^t$. Normalize δS and δI with respect to their corresponding maximal absolute value, and then fit the error structure with a 3rd-order polynomial.

4: The discrepancy in observed variable is $\Delta I = I^t - I_{obs}^t$. Calculate the structural error ΔS using the fitted error structure with proper scaling.

5: Output: The adjusted state vector at t week is $\boldsymbol{x}_{adj}^t = (S^t - \Delta S, I_{obs}^t, R_{0max}^{t-1}, R_{0min}^{t-1}, D^{t-1}, L^{t-1})^T$.

Specifically, at each observation time, the error structure between S and I is obtained by performing the breeding method from the previous observation time point. The discrepancy of the observed variable I with the observation is then computed as the difference between the I value predicted by the SIRS integration (the prior) and the true observation. After the error in S is estimated through the nonlinear error structure fitted by a 3rd-order polynomial (see Fig. 1a in the main text), both the inferred structural error in S and observed

discrepancy in I are subtracted from the prior trajectory to form an updated state vector. The pseudo-code for error correction in S-perturbed SIRS trajectory is shown in Algorithm 1.

To evaluate the accuracy of the diagnosed error ΔS, we performed an error correction procedure on a trajectory with a slightly perturbed variable S. Precisely, at 4 weeks before the peak, we imposed a $+15\%$ shock on variable S but kept the other state variables intact. After one week of model integration, we observed the exact infected population I_r, and get the predictive value I_p on the perturbed trajectory. Therefore, the error for the observed variable was the discrepancy between these two quantities: $\Delta I = I_p - I_r$. Since we have no prior knowledge of the true state variable trajectory, we performed the breeding diagnosis on the perturbed trajectory at 4 weeks prior to peak, obtaining the polynomial function, from which we can estimate the error ΔS from ΔI at 3 weeks before the peak. In the corrected trajectory, at 3 weeks prior to peak (i.e. after one week breeding the random errors imposed 4 weeks before the peak), the variables S and I were adjusted by subtracting ΔS and ΔI from S and I in the perturbed trajectory. As shown in Fig. 4, in the case of perfect observations without observational noise, a one-time correction procedure is sufficient to effectively recover the true dynamics by driving S and I close to their real values.

To generalize the above application, it is necessary to examine the performance of error correction for other combinations of parameters and initial conditions, as well as perturbations with different magnitudes at different times. Therefore, using a broad distribution of possible variable/parameter combinations, we randomly grabbed 1,000 different initial state vectors, which contained the SIRS variables S, I and parameters R_{0max}, R_{0min}, D and L. The distribution was generated by integrating 100,000 independent simulations of the SIRS model forced with New York State AH from 1972 to 2012. The unique initial set of parameters in each integration was selected with a Latin hypercube sampling strategy from the parameter space $1.3 \leq R_{0max} \leq 4$, $0.8 \leq R_{0min} \leq 1.3$, $2d \leq D \leq 7d$, $2y \leq L \leq 10y$. Then the state vectors were randomly selected from the collection of October 1 combinations.

For each combination, we perturbed the variable S at different lead times ranging from 10 weeks to 2 weeks prior to the peak. The magnitude of the shock on S lies in the interval $[-15\%, +15\%]$ with a step of 1.5%. One week after the perturbation, we used the accurate observation I_r and the perturbed predicted value I_p to diagnose and performed a one-time correction of error in S. In Fig. 5, we use a heat map to display the accuracy of the corrected

trajectory for each combination of perturbation time and magnitude. Each data point is the average result for 1,000 independent realizations of SIRS dynamics. In particular, we are interested in whether the peak timing and peak intensity can be restored. To this end, we measure the following quantities of the corrected trajectories: A) the mean error in peak timing, B) the fraction of simulations accurate for peak timing within ± 1 week, C) the mean error for peak intensity, and D) the fraction of simulations accurate for peak intensity within $\pm 25\%$. With perfect observations, peak timing can be recovered within the ± 1 week discrepancy with a probability over 90% in most cases. Peak intensity can also be restored within $\pm 25\%$ accuracy for a large collection of perturbation times and magnitudes. Notice that, the performance of the correction is asymmetric: the correction is more effective for negative perturbations on S. The reason for this asymmetry is that, given the same absolute magnitude of shocks on S, the positive shocks will create much larger trajectory deviations than negative shocks due to the nonlinear dynamics of SIRS model. Thus, it is more difficult to counterbalance the positive perturbations. Even so, the deviations can still be effectively narrowed after the correction.

Next, we performed an error correction procedure on trajectories with perturbed S (different magnitudes at different times), in the presence of observational error and uncertainty in the other state variables/parameters. For each combination, we perturbed the variable S at different lead times ranging from 10 weeks to 2 weeks prior to the peak. The magnitude of the shock on S lies in the interval $[-15\%, +15\%]$ with a step of 1.5%. At the same time, a Gaussian distributed noise with zero mean and standard deviation of 15% was added to other state variables simultaneously. The weekly error-laden observations I were generated by adding random noise drawn from a Gaussian distribution with zero mean and predefined variance 10^5. The error correction procedure on the perturbed trajectories was applied sequentially at the time of weekly observation. In Fig. 6, the peak timing and peak intensity of perturbed trajectories can be restored with high probability even though the observations are not accurate and other state variables are not perfectly known.

Supplementary Note 4. Error correction in EAKF

Suppose the observation from a synthetic outbreak is the infected population, I. Basically, for each ensemble member, the EAKF calculates the adjustment of the observed

state variable δI using a Bayesian method. The adjustments of unobservable variables and parameters are computed through their prior covariance with the observed state variable. After the update, the trajectory is constrained closer to the truth and can be integrated forward to make forecasts, as shown in Fig. 7A. In fact, the negative increment $-\delta \boldsymbol{x} = -(\delta S, \delta I, \delta R_{0max}, \delta R_{0min}, \delta D, \delta L)^T$ of the prior state can be interpreted as the estimated errors of the state variables and parameters, which are subtracted during the EAKF update.

In order to obtain a more accurate estimate of the errors in the sensitive state variable S and parameter R_{0max}, we diagnose the structural errors of S and R_{0max} using the breeding method starting from time $t-1$ (See Fig. 7B). In a realistic setting, the discrepancy of the observation is collectively caused by the errors in all the state variables/parameters. Therefore, these errors should be considered when diagnosing structural errors in S and R_{0max}. Instead of performing the diagnosis regardless of the errors in other state variables, we conducted an adjoint diagnosis by subtracting the EAKF-estimated errors from the trajectory at time $t-1$ before carrying out the breeding method. For instance, when diagnosing the structural error of S, we first removed the EAKF-estimated errors $-\delta R_{0max}$, $-\delta R_{0min}$, $-\delta D$ and $-\delta L$ from the prior trajectory (or equivalently, use the posterior of R_{0max}, R_{0min}, D and L in EAKF), and then imposed random errors on S, which evolve following the full model nonlinear dynamics, to find the error structure with observation at time t (See Fig. 7C). In operation, because the error structure is quite smooth, 20 Gaussian distributed perturbations (zero mean and standard deviation of 40%) were sufficient to capture the nonlinear relationship.

Suppose the infected population of the unperturbed trajectory at time t is I_{bred}. Note that, I_{bred} is not the prior state I_{prior} at time t predicted before EAKF adjustment, as the unperturbed trajectory in the breeding method is obtained by removing the EAKF-estimated errors from the prior trajectory. In practice, we treat the EAKF posterior observation I_{post} as the truth since it is a weighted average of the ensemble prior and the observation, which alleviates the abrupt change caused by observational error. The discrepancy of the observed variable is simply $\Delta I = I_{bred} - I_{post}$. The structural error ΔS or ΔR_{0max} can then be inferred using the 3rd-order polynomial fitting of the nonlinear error structure at time t, as shown in Fig. 7C. To prevent violent perturbations, we limit ΔS and ΔR_{0max} to within $\pm 25\%$ of their prior values at time t. Finally, we substitute for δS and δR_{0max} from the EAKF adjustment

$\delta \boldsymbol{x}$ with $-\Delta S$ and $-\Delta R_{0max}$ to form the EAKFC adjustment $\Delta \boldsymbol{x}$ (See Fig. 7D).

In the structural error correction, ΔS and ΔR_{0max} can be diagnosed in different order: i.e. the first diagnosed error can be either ΔS or ΔR_{0max}. Here we compared the performance of these two implementations for peak timing prediction in the realistic retrospective forecasts. In Fig. 8, there is no clear difference between the prediction accuracy for peak timing; however, because the dynamics are more sensitive to S, we chose to first correct errors in R_{0max}. Once ΔR_{0max} is diagnosed, it can be used in the subsequent adjoint diagnosis of ΔS, where ΔR_{0max} compensates for a further fraction of the error in the observed state variable. This order helps to avoid overly large ΔS in the subsequent diagnosis.

The pseudo-code for error correction in conjunction with EAKF is shown in Algorithm 2. Suppose the prior and posterior states in EAKF at t week are $\boldsymbol{x}^t_{prior}$ and $\boldsymbol{x}^t_{post}$. If the EAKF adjustment is $\delta \boldsymbol{x}^t$, the posterior state at t week is simply $\boldsymbol{x}^t_{post} = \boldsymbol{x}^t_{prior} + \delta \boldsymbol{x}^t$.

Algorithm 2 Error correction in EAKF

1: Input: $\boldsymbol{x}^{t-1}_{post}$, $\boldsymbol{x}^t_{prior}$, $\boldsymbol{x}^t_{post}$.

2: Diagnosis of ΔR_{0max}: Perform breeding method for R_{0max} from $t-1$ week around the trajectory $\boldsymbol{x}^{t-1}_{bred} = (S^{t-1}_{adj}, I^{t-1}_{post}, R^{t-1}_{0max_{post}}, R^t_{0min_{post}}, D^t_{post}, L^t_{post})^T$, where the EAKF-adjusted S value at week $t-1$ S^{t-1}_{adj} is obtained by integrating $\boldsymbol{x}^t_{post}$ backward for one week. $\Delta I = I^t_{bred} - I^t_{post}$. ΔR_{0max} can be solved from the fitted error structure. The updated R_{0max} at week t is $R^t_{0max_{prior}} - \Delta R_{0max}$.

3: Diagnosis of ΔS: Perform breeding method for S from week $t-1$ around the trajectory $\bar{\boldsymbol{x}}^{t-1}_{bred} = (S^{t-1}_{post}, I^{t-1}_{post}, R^t_{0max_{prior}} - \Delta R_{0max}, R^t_{0min_{post}}, D^t_{post}, L^t_{post})^T$. $\Delta I = \bar{I}^t_{bred} - I^t_{post}$. ΔS can be solved from the fitted error structure. The updated S at week t is $S^t_{prior} - \Delta S$.

4: The updated state at week t is $\boldsymbol{x}^t_{EAKFC} = (S^t_{prior} - \Delta S, I^t_{post}, R^t_{0max_{prior}} - \Delta R_{0max}, R^t_{0min_{post}}, D^t_{post}, L^t_{post})^T$.

In the above implementation, we first diagnose the structural error ΔR_{0max}. Before conducting the breeding method, we remove the EAKF-estimated errors in S, R_{0min}, D and L from the posterior state at week $t-1$ in order to compensate for the discrepancy of the observed variable I caused by these errors. This procedure is straightforward for the parameters R_{0min}, D and L: we are actually using their posterior values at week t, i.e., $R^t_{0min_{post}}$, D^t_{post} and L^t_{post}. However, for the evolving variable S, the EAKF-estimated error

in S is for week t, not $t-1$, when the breeding method starts. To find the EAKF-adjusted S at $t-1$, we integrate the posterior trajectory at week t backward for one week and use the obtained S_{adj}^{t-1} as the S value input for the breeding method. Once ΔR_{0max} is estimated, the updated R_{0max} value should be $R_{0max_{prior}}^{t} - \Delta R_{0max}$. Similarly, in the diagnosis of ΔS, the parameters R_{0min}, D and L in breeding method are also set as their posterior values. Because we have already diagnosed ΔR_{0max}, the R_{0max} value is assigned as the updated value $R_{0max_{prior}}^{t} - \Delta R_{0max}$. After the diagnosis, R_{0max} and S are updated by subtracting ΔR_{0max} and ΔS from their prior values at week t ($R_{0max_{prior}}^{t}$ and S_{prior}^{t}), while other state variables are adjusted by EAKF.

Supplementary Note 5. Application to historical influenza outbreaks

Here we report the statistical significance of the improvement provided by error correction in Fig. 2c, Fig. 3a and Fig. 3b in the main text. The traditional way to assess the confidence interval depends on an assumed probability model for the available data. However, this approach depends on a set of assumptions that often lead to inaccurate approximations. The bootstrap overcomes the above drawbacks by repeatedly estimating the desired quantity in multiple random samples of the available data. In practice, bootstrap analysis performs quite well in moderate and large data sets. In larger samples, bootstrap-estimated confidence intervals can be more accurate than confidence intervals based on standard asymptotic approximations.

From available samples of forecast accuracy for each predicted lead week, $A_1, \cdots, A_n$, we generated another set of random samples $A_1^*, \cdots, A_n^*$ by drawing the same number of observations independently with replacement. We then calculated the average accuracy $\bar{A}^*$ of the new samples. By repeating this resampling process for $m = 10^5$ times, we obtained a set of m estimates $\bar{A}_1^*, \cdots, \bar{A}_m^*$ and used this distribution to assess the likelihood of observing a specific value $\bar{A}$. Here, we first constructed the distribution of average forecast accuracy for the EAKF forecasts with 10^5 bootstrap resampling, and then calculated the p-values of corresponding EAKFC forecast accuracy according to this distribution for each predicted lead week. The p-values of EAKFC forecast accuracy for peak week, peak intensity and attack rate are listed in Table 1.

To test whether the EAKFC forecasts are significantly different from EAKF forecasts at

each week, we performed a two-sided Wilcoxon signed-rank tests on the forecast MAE of peak week, peak intensity and attack rate. The null hypothesis is that the difference between the matched samples (EAKFC and EAKF MAE in each forecast) comes from a distribution whose median is zero. If p-values are lower than a certain significance level (e.g., 0.05), the difference between these two forecasts is statistically significant. We report the test results for the forecasts of 30 consecutive weeks starting from Oct. 1st in Table 2.

It was previously found that the forecast skill of the EAKF could be discriminated by the spread of ensemble predictions [13]. The same relationship between prediction accuracy and ensemble spread also holds for EAKFC and EAKFIC: decreased ensemble variance indicates increased forecast certainty. To better illustrate this, we stratified retrospective forecasts based on the ensemble variance of peak timing and compared their accuracy in Fig. 9. Grouped by how many weeks in the future the peak is predicted, predictions for 95 cities in the United States (100 independent forecasts generated, each with a 300-member ensemble, for each city and each week during each season) were first sorted in ascending order of ensemble variance for peak timing, and then the 50% of forecasts with lower spread were selected to compare prediction accuracy against all forecasts. We present peak timing accuracy (± 1 week) and the predictive probability of the real peak (± 1 week) in Fig. 9(A-B) for the EAKF, EAKFC and EAKFIC. Both measures of prediction certainty are improved for the stratified group with smaller ensemble spread. Moreover, the stratified EAKFC and EAKFIC predictions exhibit greater forecast accuracy than the stratified EAKF forecasts, confirming the effectiveness of structural error correction.

For the individual influenza seasons from 2003-2004 to 2013-2014, excluding 2008-2009 and 2009-2010 pandemic seasons, we report the reduction of forecast MAE due to error correction for peak timing, peak intensity and attack rate in Table 3. Positive values in Table 3 indicate reduced MAE achieved by error correction. The results are averaged over the forecasts for all 95 cities. For the predicted lead time from 6 weeks to 0 week, the error correction procedure effectively decreases forecast MAE in most cases (see the positive values in Table 3).

To further examine the forecast quality with error growth correction, we can explore how the realistic observations distribute with respect to the predicted values. If the forecasts are providing a good estimate of the truth, the observations (single realization of a stochastic process) should be normally distributed around the predictions with zero mean-

that is an accurate forecast. If on the other hand the mean error is non-zero, there remains bias/inaccuracy in the prediction. By looking at the scatter of observations around many predictions, we can obtain an estimate of that inaccuracy.

In Fig. 10-12, we show the distributions of the distance from realistic observations (peak week, peak intensity and attack rate, respectively) to the predicted values across all 95 cities and 9 seasons. In particular, we first grouped the forecasts according to their predicted lead to peak from 6 weeks to -2 weeks, and then plotted the distribution of the discrepancy of true observations from corresponding predictions within each category, for both EAKF and EAKFC predictions. Compared with the distribution of EAKF, the EAKFC forecast has less bias than the EAKF for all three targets. That is, by diagnosing nonlinear error growth, we can obtain a better estimate of forecast initial conditions and reduce forecast inaccuracy.

Supplementary Note 6. Iterative application of error correction

In a naive iterative application of error correction, when the ensemble is not well trained by EAKF, error correction may introduce large errors that can propagate following updates. To avoid such improper updating in the EAKFIC, we control the condition for the iterative application of error correction. A simple and practical strategy is to update the prior trajectories with error correction only if the EAKF predicted lead time is smaller than a specified threshold. We tested various choices of lead time threshold (3-10 weeks) and display the results in Fig. 13. Per this comparison, we selected an 8-week threshold for presentation in the main text, although all threshold choices improved peak timing accuracy at long lead times.

Supplementary Note 7. Error correction in a stochastic model

In previous implementation of error correction, we used a deterministic SIRS model to simulate influenza dynamics. To address whether error growth correction would similarly improve forecast accuracy for a stochastic model, we also performed error growth correction and retrospective forecast using a stochastic version of the SIRS model. In this form, new infection, recovery and immunity loss populations were generated by a Poisson process with mean values determined by the model equations. This stochastic model form adds another

level of uncertainty to the model system. In Fig. 14, the average forecast accuracy of peak timing, peak intensity and attack rate is presented. As for the deterministic model, error correction with the stochastic model improves forecast accuracy for all 3 metrics in advance of the peak. In the deterministic model, uncertainty is derived from model misspecification and error in the initial conditions, whereas in the stochastic model, additional uncertainty is derived from random noise.

In a dynamical model, random noise can be critical in defining the long-term predictability of the system. For instance, with enough stochasticity, a simple nonlinear deterministic model can generate complex time series that look chaotic [14]. Before the 1990s, there was great interest in understanding irregular biological dynamics through chaos theory. However, recent advances indicate that such dynamics can be better explained by nonlinearity plus stochasticity, or "noise-induced chaos" [14]. For the purposes of forecasting, a deterministic model can predict a system's short term dynamics with high accuracy, whereas the long term deterministic dynamics may be very different from those generated with random noises. Given the role of random noise, good representation of noise may be important for understanding error growth when generating longer-term forecasts (e.g. more than one season).

In this work, the forecast scope is limited to a relatively short range of up to several months. At this time scale, the behaviour of the observations is dominated by signal produced by influenza outbreak transmission dynamics. Random noise in the model caused by stochasticity may grow during integration, but will not generally produce qualitative differences over such a short time interval. Indeed, complex behaviors such as chaos are unlikely to appear in the weakly nonlinear SIRS model over such short time periods, particularly over the week-long time period we use for diagnosing error growth. Therefore, error correction predominantly makes use of the nonlinear initial error growth pattern. Further, note that the breeding method diagnosis of this nonlinear initial error growth pattern performed on both deterministic and stochastic models produced similar error growth patterns. That error correction based on these diagnoses improves forecast accuracy for both stochastic and deterministic model structures indicates that nonlinear initial error growth is a factor corrupting forecasts made with either model structure.

Supplementary Note 8. Sensitivity to the scaling parameter

To examine the sensitivity of forecast results to the scaling parameter, we performed retrospective forecasts with different scaling parameter γ, ranging from 0.5 to 1.5. For peak timing, peak intensity and attack rate, we report the improvement of EAKFC accuracy over EAKF in Table 4 at each predicted lead week. A positive value means EAKFC outperforms EAKF, while a negative value implies the opposite. From Table 4, it is concluded that the improvement achieved by error correction is not particularly sensitive to the specific choice of scaling parameter. Note that the scaling parameter γ is important for both forecasts and the scientific interpretation of the model. Additional care might be required if one is interested in metrics other than prediction quality.

Metric	10 week	9 week	8 week	7 week	6 week	5 week	4 week	3 week	2 week	1 week	0 week
Pw	$< 10^{-5}$	$< 10^{-5}$	$< 10^{-5}$	$< 10^{-5}$	$< 10^{-5}$	$< 10^{-5}$	$< 10^{-5}$	$< 10^{-5}$	$< 10^{-5}$	$< 10^{-5}$	$< 10^{-5}$
Pi	10^{-5}	$< 10^{-5}$	$< 10^{-5}$	0.4184	$< 10^{-5}$	$< 10^{-5}$	$< 10^{-5}$	$< 10^{-5}$	$< 10^{-5}$	$< 10^{-5}$	$< 10^{-5}$
Ar	0.0754	0.4913	0.0829	0.0013	$< 10^{-5}$	$< 10^{-5}$	$< 10^{-5}$	$< 10^{-5}$	$< 10^{-5}$	$< 10^{-5}$	$< 10^{-5}$

SUPPLEMENTARY TABLE 1. We report the p-values of EAKFC forecast accuracy for peak week (Pw), peak intensity (Pi) and attack rate (Ar) obtained by bootstrap analysis. We first constructed the distribution of average forecast accuracy for the EAKF forecasts with 10^5 bootstrap resampling, and then calculated the p-values of corresponding EAKFC forecast accuracy according to this distribution for each predicted lead week.

Metric	1 week	2 week	3 week	4 week	5 week	6 week	7 week	8 week	9 week	10 week
Pw	0.5269	$< 10^{-5}$	$< 10^{-5}$	$< 10^{-5}$	$< 10^{-5}$	$< 10^{-5}$	$< 10^{-5}$	$< 10^{-5}$	$< 10^{-5}$	$< 10^{-5}$
Pi	$< 10^{-5}$	$< 10^{-5}$	$< 10^{-5}$	$< 10^{-5}$	$< 10^{-5}$	$< 10^{-5}$	$< 10^{-5}$	$< 10^{-5}$	$< 10^{-5}$	$< 10^{-5}$
Ar	$< 10^{-5}$	$< 10^{-5}$	$< 10^{-5}$	$< 10^{-5}$	$< 10^{-5}$	$< 10^{-5}$	$< 10^{-5}$	$< 10^{-5}$	$< 10^{-5}$	$< 10^{-5}$

Metric	11 week	12 week	13 week	14 week	15 week	16 week	17 week	18 week	19 week	20 week
Pw	$< 10^{-5}$	$< 10^{-5}$	$< 10^{-5}$	$< 10^{-5}$	$< 10^{-5}$	$< 10^{-5}$	$< 10^{-5}$	$< 10^{-5}$	$< 10^{-5}$	$< 10^{-5}$
Pi	$< 10^{-5}$	0.8881	0.0002	$< 10^{-5}$	$< 10^{-5}$	$< 10^{-5}$	$< 10^{-5}$	$< 10^{-5}$	$< 10^{-5}$	$< 10^{-5}$
Ar	$< 10^{-5}$	$< 10^{-5}$	$< 10^{-5}$	$< 10^{-5}$	$< 10^{-5}$	$< 10^{-5}$	$< 10^{-5}$	$< 10^{-5}$	$< 10^{-5}$	$< 10^{-5}$

Metric	21 week	22 week	23 week	24 week	25 week	26 week	27 week	28 week	29 week	30 week
Pw	$< 10^{-5}$	$< 10^{-5}$	$< 10^{-5}$	0.0018	$< 10^{-5}$	$< 10^{-5}$	$< 10^{-5}$	0.0439	$< 10^{-5}$	0.7576
Pi	$< 10^{-5}$	$< 10^{-5}$	$< 10^{-5}$	0.0877	0.6787	1	0.0003	1	1	1
Ar	$< 10^{-5}$	$< 10^{-5}$	0.2332	0.9316	$< 10^{-5}$	$< 10^{-5}$	0.0465	0.0282	0.8342	$< 10^{-5}$

SUPPLEMENTARY TABLE 2. We present the p-values of forecast MAE for peak week (Pw), peak intensity (Pi) and attack rate (Ar) obtained using two-sided Wilcoxon signed-rank tests at each forecast week, beginning the first week of October.

Season	Metric	6 week	5 week	4 week	3 week	2 week	1 week	0 week
	Pw	1.06(34.0%)	0.63(22.3%)	0.24(9.8%)	0.18(8.8%)	0.19(9.4%)	0.09(4.7%)	0.10(6.2%)
2013	Pi	62(2.2%)	238(8.4%)	618(20.8%)	518(18.6%)	-273(-11.0%)	-100(-5.1%)	121(19.7%)
	Ar	1585(10.0%)	2106(13.3%)	3615(22.9%)	2304(15.9%)	-212(-1.6%)	-44(-0.4%)	387(6.0%)
	Pw	0.76(17.1%)	0.68(14.6%)	1.22(24.8%)	1.63(34.4%)	1.25(29.5%)	1.09(29.4%)	0.30(17.6%)
2012	Pi	131(1.6%)	746(8.8%)	2049(23.7%)	2362(28.6%)	1102(15.3%)	1222(18.0%)	631(18.7%)
	Ar	5179(8.6%)	9379(15.5%)	13871(23.1%)	12786(22.6%)	9108(17.3%)	6864(14.3%)	4780(13.3%)
	Pw	2.07(16.6%)	3.44(29.0%)	3.93(36.1%)	3.61(37.8%)	2.13(29.3%)	-0.72(-15.5%)	-1.25(-38.7%)
2011	Pi	14(1.4%)	49(4.9%)	-16(-1.6%)	77(7.0%)	328(25.4%)	53(5.5%)	-100(-18.3%)
	Ar	-508(-5.3%)	739(7.9%)	818(8.7%)	1804(16.1%)	4132(33.1%)	1388(15.5%)	504(7.8%)
	Pw	2.46(29.3%)	2.53(31.9%)	2.41(34.3%)	1.88(30.4%)	1.06(20.3%)	0.85(20.9%)	0.08(4.2%)
2010	Pi	297(13.1%)	225(10.2%)	455(20.5%)	521(23.7%)	113(6.0%)	81(4.5%)	16(1.4%)
	Ar	3793(19.5%)	3416(18.4%)	5733(31.1%)	6053(33.6%)	3024(19.7%)	1613(12.2%)	1207(11.8%)
	Pw	2.11(26.9%)	3.26(43.3%)	3.10(47.9%)	1.72(34.9%)	0.52(14.0%)	0.10(3.0%)	-0.86(-56.5%)
2007	Pi	272(7.0%)	400(9.8%)	578(13.8%)	1009(22.9%)	870(21.9%)	156(5.2%)	-407(-31.2%)
	Ar	78(0.4%)	2329(10.2%)	5068(20.7%)	7926(30.7%)	5454(25.0%)	1573(9.7%)	-1889(-19.7%)
	Pw	2.57(30.7%)	2.62(32.8%)	2.60(36.7%)	1.11(20.5%)	0.58(12.8%)	0.72(17.8%)	0.27(10.2%)
2006	Pi	-32(-2.6%)	220(16.6%)	181(12.4%)	242(16.0%)	-380(-30.4%)	97(7.4%)	143(18.1%)
	Ar	-363(-4.1%)	694(7.6%)	752(7.7%)	378(3.7%)	-2759(-29.4%)	1086(9.8%)	1208(15.7%)
	Pw	1.09(15.5%)	1.01(15.1%)	1.16(18.7%)	0.83(16.6%)	0.17(4.1%)	-0.62(-18.5%)	-0.70(-31.1%)
2005	Pi	249(17.4%)	382(27.8%)	109(9.9%)	-166(-16.3%)	-299(-26.6%)	161(13.5%)	-69(-13.9%)
	Ar	1725(15.7%)	2059(19.5%)	1140(12.3%)	-464(-5.5%)	-1053(-11.6%)	1013(11.6%)	391(6.3%)
	Pw	2.15(28.4%)	2.17(30.9%)	2.29(35.9%)	1.56(29.5%)	0.58(14.0%)	-0.79(-22.6%)	-0.60(-25.5%)
2004	Pi	160(8.7%)	-101(-5.6%)	190(9.6%)	-18(-0.8%)	326(14.4%)	32(1.8%)	-161(-21.0%)
	Ar	748(6.3%)	-612(-5.3%)	1640(12.6%)	920(6.8%)	1766(13.2%)	-1036(-10.6%)	-1206(-19.7%)
	Pw	-0.69(-61.8%)	-0.58(-48.2%)	-0.24(-17.7%)	0.17(11.1%)	0.31(23.1%)	0.08(7.2%)	0.06(6.9%)
2003	Pi	1077(15.4%)	1761(25.8%)	2074(33.9%)	1447(28.9%)	743(16.5%)	477(10.7%)	579(38.4%)
	Ar	4495(19.7%)	5122(23.3%)	4417(22.2%)	4449(23.9%)	2033(11.2%)	-506(-2.8%)	856(7.0%)

SUPPLEMENTARY TABLE 3. EAKFC improvement of forecast MAE versus EAKF for different seasons. The results for peak week (Pw), peak intensity (Pi) and attack rate (Ar) are averaged over all 95 cities in each season. The predicted lead time to peak is obtained from the EAKF and EAKFC forecasts, respectively. Numbers in the parenthesis are the percentage of EAKF MAE reduced by error correction. In the table, positive values represent the reduction (improvement) of MAE achieved by EAKFC.

Scaling	Metric	10 week	9 week	8 week	7 week	6 week	5 week	4 week	3 week	2 week	1 week	0 week
0.5	Pw	0.0270	0.0666	0.0657	0.0460	0.0459	0.0815	0.1067	0.1500	0.0961	0.0267	-0.0243
	Pi	-0.0769	-0.0541	-0.0590	-0.0532	0.0027	0.0172	0.0172	0.0404	0.0535	0.0790	-0.0502
	Ar	-0.0881	-0.0079	0.0052	0.0007	0.0813	0.0454	0.0044	-0.0252	-0.0326	-0.0171	-0.0600
0.6	Pw	-0.0355	0.0976	0.0559	0.0266	0.0558	0.0786	0.1176	0.1662	0.1356	0.0451	-0.0008
	Pi	-0.0252	-0.0715	-0.0126	0.0099	0.0081	0.0494	0.0282	0.0493	0.0994	0.0528	-0.0184
	Ar	0.0427	-0.0113	-0.0074	0.0743	0.0791	0.0960	0.0575	0.0391	-0.0092	-0.0169	-0.0010
0.7	Pw	0.0059	0.1047	0.0495	0.0404	0.0546	0.0749	0.1026	0.1612	0.1705	0.0742	-0.0027
	Pi	0.0361	0.0046	0.0083	0.0042	0.0375	0.0248	0.0250	0.0606	0.1065	0.0794	-0.0003
	Ar	-0.0124	-0.0466	0.0337	0.1144	0.0888	0.0568	0.0333	0.0701	0.0222	0.0059	0.0189
0.8	Pw	0.0247	0.0287	0.0401	0.0515	0.0543	0.0544	0.1078	0.1693	0.1878	0.1180	0.0106
	Pi	-0.0087	0.0104	-0.0109	0.0068	0.0302	0.0460	0.0326	0.0593	0.0896	0.0939	0.0126
	Ar	0.0170	0.0598	0.0555	0.0970	0.0691	0.0722	0.0898	0.1134	0.0825	0.0718	0.0126
0.9	Pw	0.0766	0.0184	0.0433	0.0472	0.0480	0.0554	0.0854	0.1438	0.1940	0.1270	0.0286
	Pi	0.0032	-0.0151	0.0063	0.0132	0.0346	0.0366	0.0463	0.0621	0.1107	0.0962	0.0273
	Ar	-0.0589	0.0586	0.0827	0.1265	0.0949	0.0732	0.1296	0.1238	0.0964	0.0897	0.0497
1.0	Pw	0.0101	0.0835	0.0325	0.0236	0.0596	0.0491	0.0662	0.1643	0.1806	0.1309	0.0334
	Pi	0.0144	0.0941	0.0774	-0.0025	0.0332	0.0408	0.0715	0.0641	0.1173	0.0944	0.0378
	Ar	0.0370	0.1769	0.1409	0.0884	0.0857	0.0845	0.1079	0.1442	0.1224	0.0927	0.0713
1.1	Pw	0.0428	0.0471	0.0624	0.0292	0.0329	0.0450	0.0691	0.1385	0.1781	0.1214	0.0375
	Pi	-0.0440	-0.0226	0.0062	0.0740	0.0217	0.0239	0.0729	0.0763	0.1092	0.0891	0.0370
	Ar	0.0162	0.0736	0.1714	0.1497	0.0374	0.0557	0.1184	0.1545	0.1387	0.0737	0.0742
1.2	Pw	0.0130	0.0602	0.0365	0.0420	0.0663	0.0520	0.0535	0.1228	0.1673	0.1285	0.0376
	Pi	-0.0097	0.0659	0.0257	0.0188	0.0254	0.0349	0.0775	0.0783	0.1028	0.0771	0.0329
	Ar	-0.0040	0.1826	0.1672	0.0919	0.0816	0.0456	0.1264	0.1429	0.1330	0.1055	0.0750
1.3	Pw	0.1090	-0.0159	0.0387	0.0289	0.0350	0.0331	0.0642	0.1208	0.1662	0.1365	0.0429
	Pi	0.0181	0.0457	0.0336	0.0450	0.0314	0.0184	0.0406	0.0828	0.1140	0.0932	0.0409
	Ar	0.0322	0.0949	0.1415	0.1198	0.0406	0.0586	0.1191	0.1485	0.1539	0.1188	0.0734
1.4	Pw	0.0529	0.0896	0.0517	0.0245	0.0508	0.0459	0.0490	0.1084	0.1640	0.1312	0.0382
	Pi	0.0094	0.0770	0.0307	0.0290	0.0330	0.0338	0.0762	0.0873	0.1181	0.0854	0.0381
	Ar	-0.0032	0.0902	0.0685	0.0795	0.0656	0.0614	0.1220	0.1437	0.1582	0.1021	0.0593
1.5	Pw	0.0096	0.1105	0.0268	0.0317	0.0505	0.0513	0.0382	0.1101	0.1406	0.1393	0.0424
	Pi	0.0810	0.0017	0.0360	0.0233	0.0192	0.0211	0.0546	0.0784	0.1099	0.1075	0.0443
	Ar	0.0147	0.0859	0.1020	0.0833	0.0462	0.0208	0.0964	0.1592	0.1646	0.1103	0.0725

SUPPLEMENTARY TABLE 4. Forecast accuracy improvement of EAKFC over EAKF for peak week (Pw), peak intensity (Pi) and attack rate (Ar), with different scaling parameter γ from 0.5 to 1.5. For predicted leads between 10 weeks to 0 week, we report the difference of forecast accuracy between EAKFC and EAKF. Positive values indicate EAKFC outperforms EAKF.

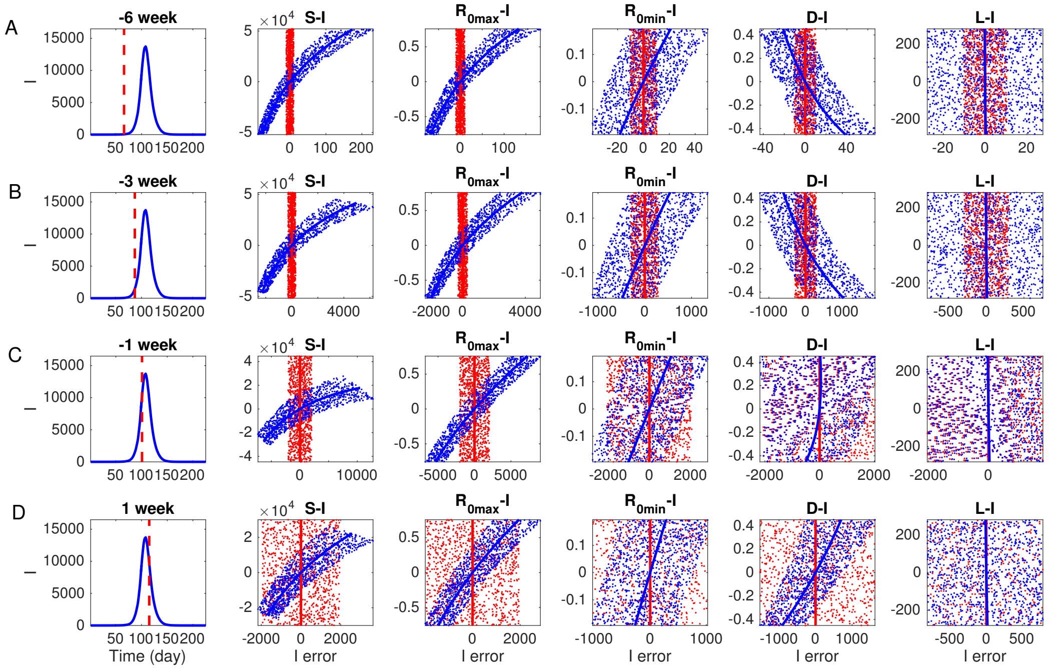

SUPPLEMENTARY FIG. 1. Error structure in the humidity-forced SIRS model at different times. For the SIRS model with parameters $L = 3.86y$, $D = 2.27d$, $R_{0max} = 3.79$, $R_{0min} = 0.97$ and initial condition $S(0) = 250,000$, $I(0) = 1$, we impose 1,000 random perturbations on both the unobserved state variable and parameters (S, R_{0max}, R_{0min}, D, L) and the observed variable I at the following times: 6 weeks, 3 weeks, 1 week prior to peak and 1 week after peak. The initial perturbations are uniformly distributed in the region $[-20\%, 20\%] \times [-20\%, 20\%]$, displayed by the red dots. After one week, we present the bred errors as blue dots. The solid red and blue lines show the cases in which we only perturb the unobserved state variable or parameters but keep the observed variable I unchanged.

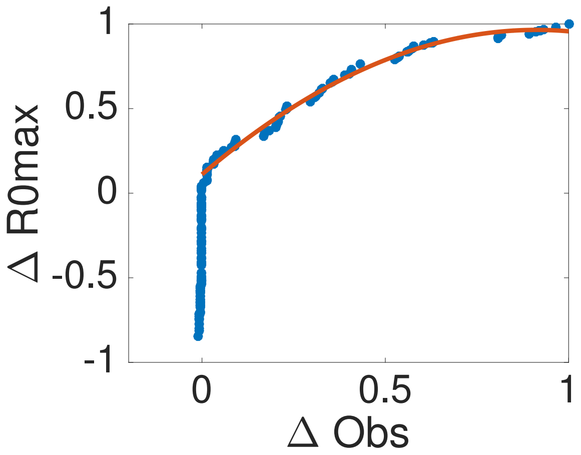

SUPPLEMENTARY FIG. 2. Error structure near the critical point. Error structure between the unobserved parameter R_{0max} and weekly incidence following perturbations imposed 3 weeks prior to peak in a synthetic outbreak ($N = 10^5$, $S(0) = 0.5N$, $I(0) = 1$, $L = 3.86y$, $D = 2.27d$, $R_{0max} = 3.0$, $R_{0min} = 0.97$).

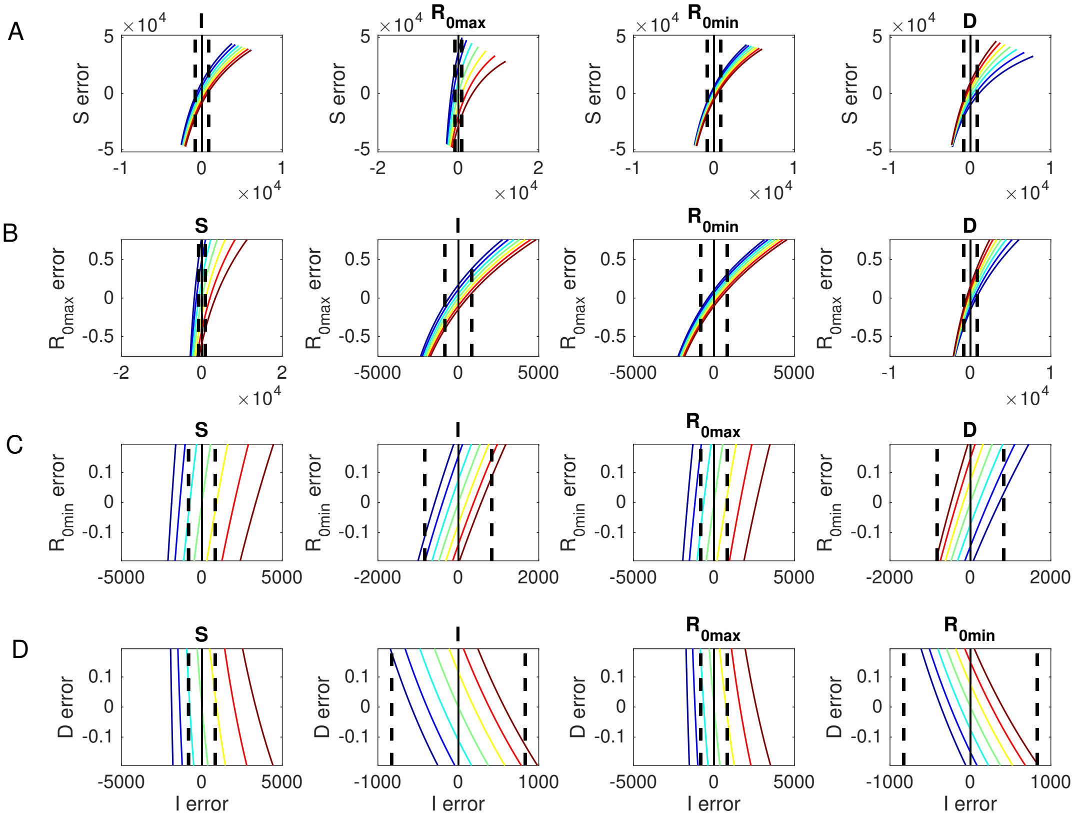

SUPPLEMENTARY FIG. 3. Robustness of error structure in the presence of error among other state variables at 3 weeks prior to peak. The bred error structures between S, R_{0max}, R_{0min}, D and the observed variable I in the presence of additional perturbations on other state variables are displayed in A-D. The pa-rameters and initial condition of the SIRS model are set as in Fig. 1. The additional perturbations on other state variables are imposed 3 weeks before the peak. The y-axis is the variable/parameter whose robustness of error structure is examined, while the title of each panel indicates the state variable that is additionally perturbed. From the blue to red curve, the magnitude of perturbation on the additionally perturbed state variable ranges from -15% to $+15\%$ with a 5% interval. Dif-ferent coloured lines represent the error structure between the y-axis variable/ parameter and the observed variable as a function of additional errors in the title variable/ parameter. The vertical dash lines mark the $\pm 20\%$ error boundary of observation.

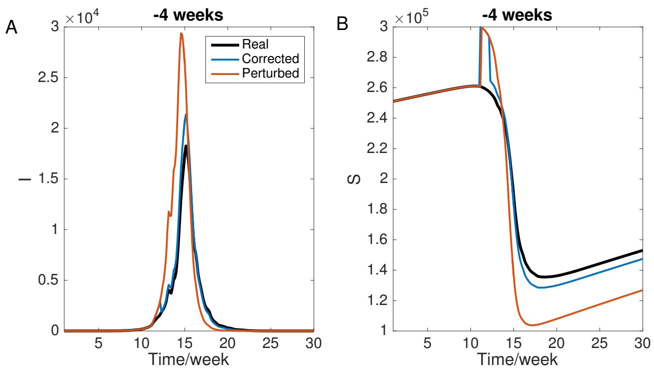

SUPPLEMENTARY FIG. 4. Correction of diagnosed errors in S in a simulated outbreak with perfect observation. One-time correction of the $+15\%$ error in S imposed 4 weeks before the peak, with perfect observation I. The real, perturbed and corrected curves are shown in different colors. The SIRS simulation is run with parameters $L = 3.86y$, $D = 2.27d$, $R_{0max} = 3.79$, $R_{0min} = 0.97$ and initial condition $S(0) = 250,000$, $I(0) = 1$.

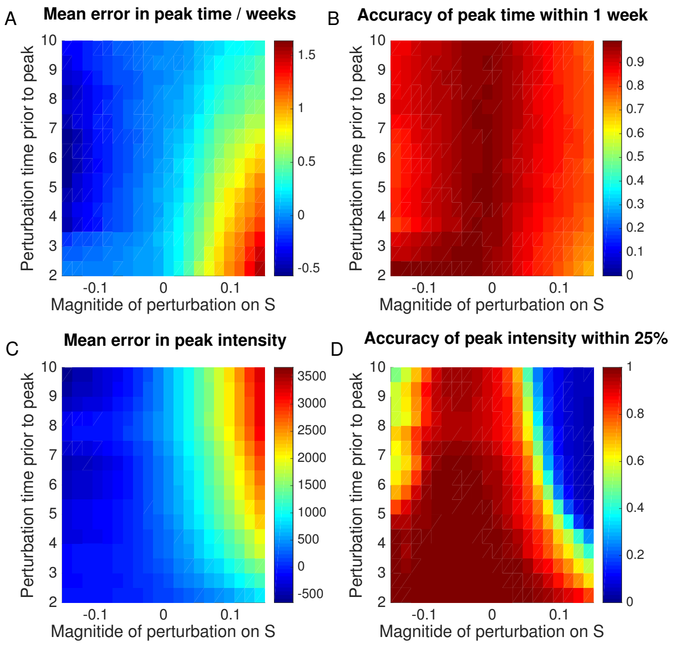

SUPPLEMENTARY FIG. 5. One-time correction of diagnosed errors in S in the presence of perfect observations. For SIRS model simulations using 1,000 different combinations of parameters and initial conditions, we impose shocks ranging from -15% to $+15\%$ on S at different times (2 to 10 weeks) before peak. One-time error correction of S as diagnosed by the breeding method is then performed one week later. For each combination of perturbation magnitude and perturbation time, we use a heat map to present the errors for peak timing and intensity of the corrected trajectories compared to the truth: A the mean error in peak timing, B the fraction of simulations accurate for peak timing within ± 1 week, C the mean error for peak intensity, and D the fraction of simulations accurate for peak intensity within $\pm 25\%$.

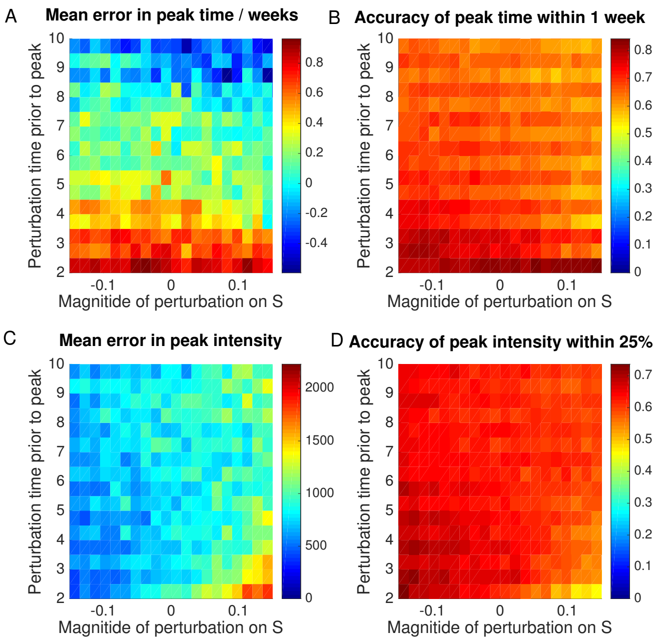

SUPPLEMENTARY FIG. 6. Correction of diagnosed errors in S in the case of noisy observations and perturbed state variables. The noises applied to I, R_{0max}, R_{0min}, D and L follow a Gaussian distribution with zero mean and standard deviation of 15%. The observational error is also Gaussian distributed with zero mean and variance of 10^5. For each combination of perturbation magnitude and perturbation time, we use a heat map to present the errors for peak timing and intensity of the corrected trajectories compared to the truth: A the mean error in peak timing, B the fraction of simulations accurate for peak timing within ±1 week, C the mean error for peak intensity, and D the fraction of simulations accurate for peak intensity within ±25%.

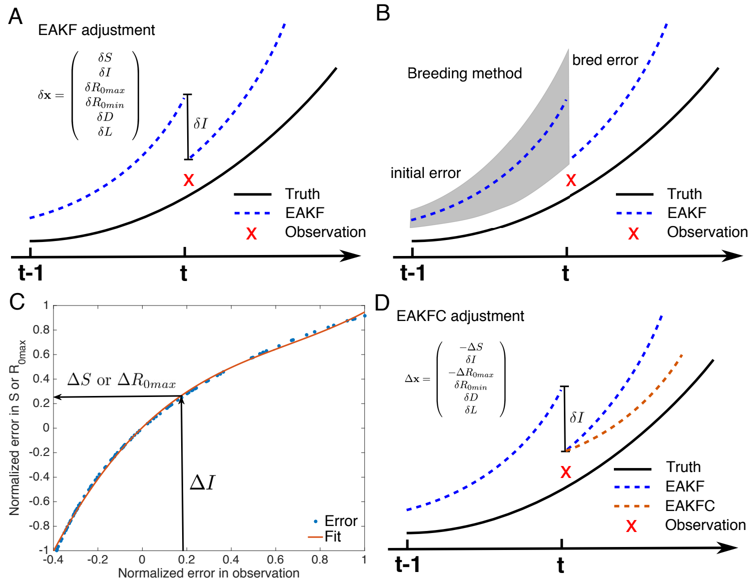

SUPPLEMENTARY FIG. 7. Schematic illustration of EAKFC process. A At time t, the EAKF adjusts the prior tra-jectory in conjunction with the observation at t, after which the posterior trajectory is constrained closer to the truth. B Application of the breeding method on the trajectory from time $t-1$. The initial random errors imposed at time $t-1$ evolve following the full nonlinear dynamics until t. C Use of the obtained bred error structure at time t to infer errors in R_{0max} (ΔR_{0max}) and S (ΔS) sequentially. The error of the observed state variable ΔI is obtained by comparing the observation with the posterior observation adjusted by EAKF. The nonlinear error structure is estimated using a 3rd-order polynomial. D Substitution of δS and δR_{0max} into the EAKF adjustment $\delta \mathbf{x}$ by $-\Delta S$ and $-\Delta R_{0max}$ to form the EAKFC adjustment $\Delta \mathbf{x}$. Then the prior trajectory at time t is adjusted by $\Delta \mathbf{x}$ to make predictions.

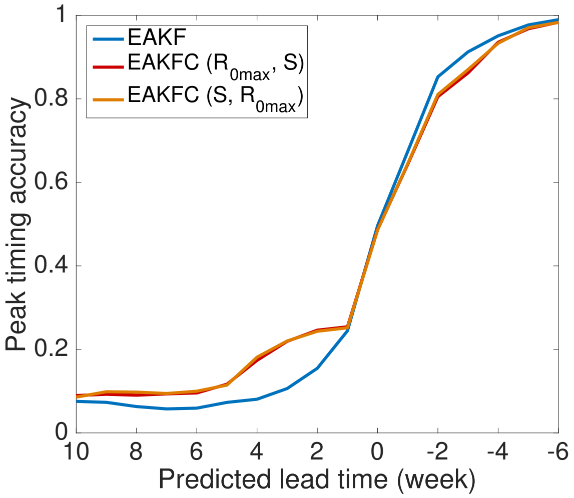

SUPPLEMENTARY FIG. 8. Effect of the correction order of S and R_{0max} on peak timing prediction. EAKFC pre-dictions with different correction order for realistic influenza outbreaks in 95 US cities during the 2003-2004 to 2013-2014 seasons (excluding the 2008-2009 and 2009-2010 pandemic outbreaks) are performed. Comparison between these two predictions for peak timing accuracy (± 1 week) is displayed.

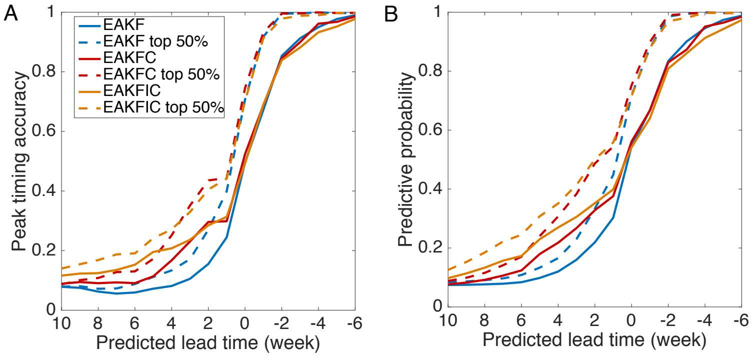

SUPPLEMENTARY FIG. 9. Prediction quality is improved by stratification using the ensemble variance of predicted peak timing. We consider EAKF, EAKFC and EAKFIC predictions for realistic influenza out-breaks in 95 US cities during the 2003-2004 to 2013-2014 seasons, excluding the 2008-2009 and 2009-2010 pandemic outbreaks. For each predicted lead time, we rank the predictions based on their ensemble variance of peak timing in an ascending order and select the 50% with lower ensem-ble variance. Comparisons between the stratified (dash lines) and overall predictions (solid lines) for average peak timing accuracy (±1 week) and predictive probability of real peak (±1 week) are shown in A-B, respectively.

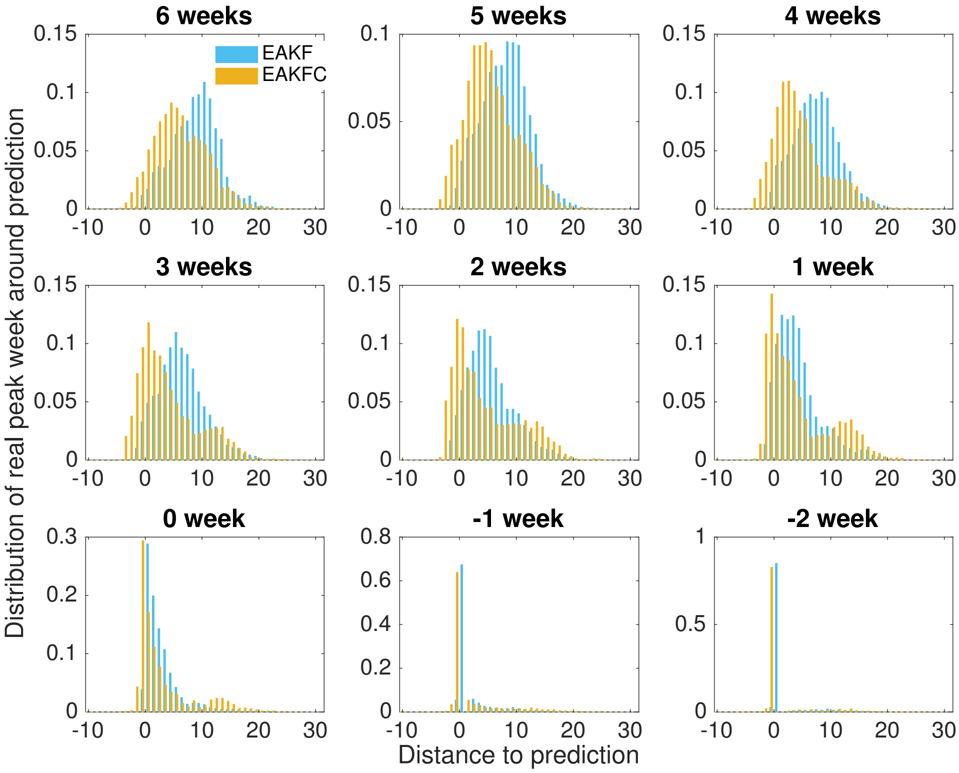

SUPPLEMENTARY FIG. 10. Distributions of the distance from the observed peak week to the predicted value. For predictions with a given predicted lead from 6 weeks to -2 weeks, the distributions of the observed peak week with respect to the predicted values (x-axis is the value of observed peak week minus predicted peak week) across all 95 cities and 9 seasons are displayed, for both EAKF and EAKFC forecasts.

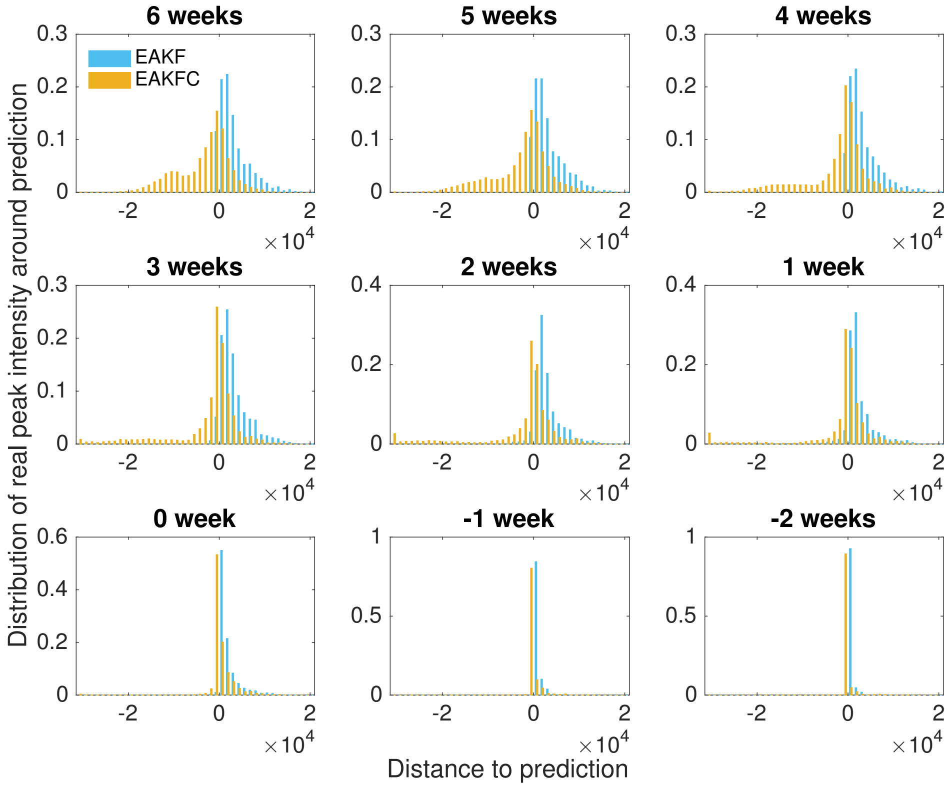

SUPPLEMENTARY FIG. 11. Same analysis for peak intensity as in Fig. 10.

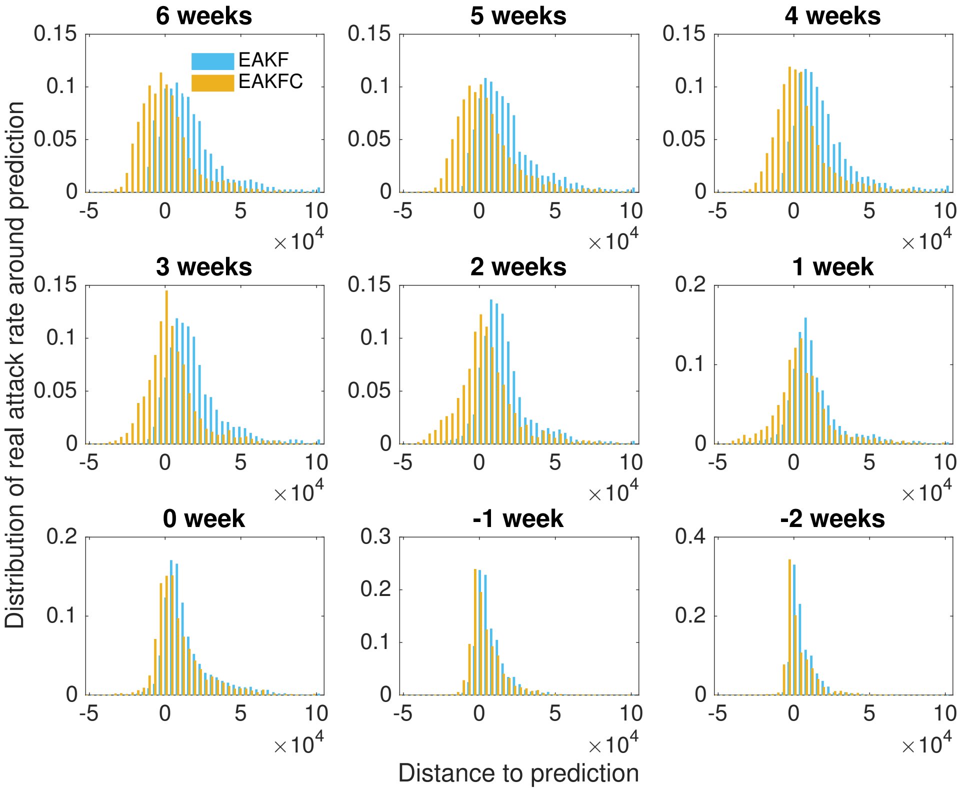

SUPPLEMENTARY FIG. 12. Same analysis for attack rate as in Fig. 10.

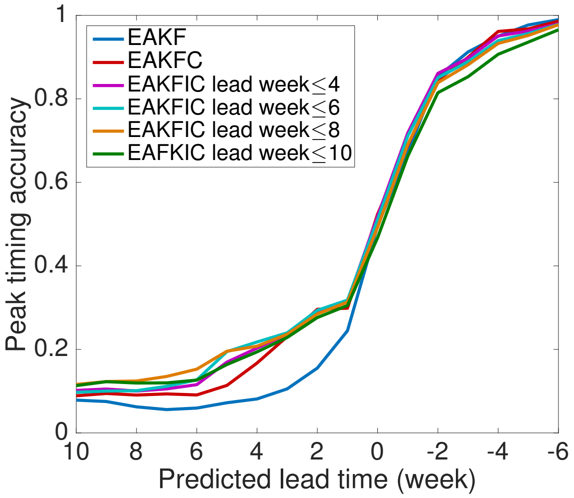

SUPPLEMENTARY FIG. 13. Performance of different thresholds in the iterative application of error correction. In EAKFIC, prior trajectories are updated using error correction only if the EAKF predicted lead time is smaller than a specified threshold. Peak timing forecast accuracy for retrospective forecasts with thresholds of 4, 6, 8 10 weeks are compared.

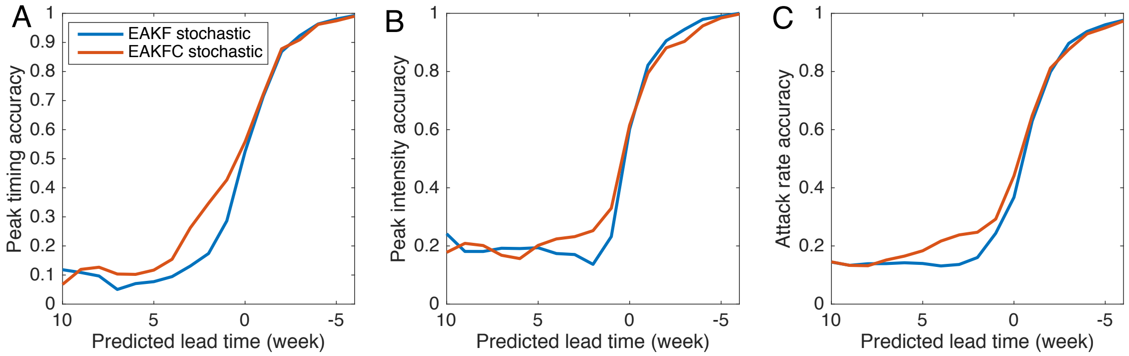

SUPPLEMENTARY FIG. 14. Forecast accuracy with a stochastic humidity-driven SIRS model. We performed retro-spective forecasts with a stochastic humidity-driven SIRS model for 95 cities in the United States for the 2003-2004 through 2013-2014 seasons, excluding the 2008-2009 and 2009-2010 pandemic seasons. The average forecast accuracy of peak timing (A), peak intensity (B) and attack rate (C) is compared for EAKF and EAKFC.

SUPPLEMENTARY REFERENCES

[1] Shaman J, Pitzer VE, Viboud C, Grenfell BT, Lipsitch M (2010) Absolute humidity and the seasonal onset of influenza in the continental US. *PLoS Biol* 8(2):e1000316.

[2] Shaman J, Kohn MA (2009) Absolute humidity modulates influenza survival, transmission and seasonality. *Proc Natl Acad Sci USA* 106(9):3243-3248.

[3] Toth Z, Kalnay E (1993) Ensemble forecasting at NMC: The generation of perturbations. *B Am Meteorol Soc* 74(12):2317-2330.

[4] Toth Z, Kalnay E (1997) Ensemble forecasting at NCEP and the breeding method. *Mon Weather Rev* 125(12):3297-3319.

[5] Kuznetsov, Y. A. & Piccardi, C. Bifurcation analysis of periodic SEIR and SIR epidemic models. *J. Math. Biol.* **32,** 109-121 (1994).

[6] Lagorio, C. et al. Quarantine-generated phase transition in epidemic spreading. *Phys. Rev. E* **83,** 026102 (2011).

[7] Aguiar, M., Kooi, B. & Stollenwerk, N. Epidemiology of dengue fever: A model with temporary cross-immunity and possible secondary infection shows bifurcations and chaotic behaviour in wide parameter regions. *Math. Mod. Nat. Phen..* **3,** 48-70 (2008).

[8] Xiao, H. et al. Animal reservoir, natural and socioeconomic variations and the transmission of hemorrhagic fever with renal syndrome in Chenzhou, China, 20062010. *PLoS Negl. Trop. Dis.* **8,** e2615 (2014).

[9] Bolker, B. M. & Grenfell, B. T. Chaos and biological complexity in measles dynamics. *Proc. R. Soc. B* **251,** 75-81 (1993).

[10] Grossman, Z. Oscillatory phenomena in a model of infectious diseases. *Theor. Popul. Biol.* **18,** 204-243 (1980).

[11] Stone, L., Olinky, R. & Huppert, A. Seasonal dynamics of recurrent epidemics. *Nature* **446,** 533-536 (2007).

[12] Axelsen, J. B., Yaari, R., Grenfell, B. T. & Stone, L. Multiannual forecasting of seasonal influenza dynamics reveals climatic and evolutionary drivers. *Proc. Natl. Acad. Sci. USA* **111,** 9538-9542 (2014).

[13] Shaman J, Karspeck A (2012) Forecasting seasonal outbreaks of influenza. *Proc Natl Acad Sci USA* 109(50):20425-20430.

[14] Ellner, S. P. & Turchin, P. When can noise induce chaos and why does it matter: a critique. *Oikos* **111,** 620-631 (2005).
